# Augmented reality for the virtual dissection of white matter pathways

**DOI:** 10.1007/s00701-020-04545-w

**Published:** 2020-10-07

**Authors:** Sebastian Ille, Ann-Katrin Ohlerth, David Colle, Henry Colle, Olga Dragoy, John Goodden, Pierre Robe, Adrià Rofes, Emmanuel Mandonnet, Erik Robert, Djaina Satoer, Catarina Pessanha Viegas, Evy Visch-Brink, Martine van Zandvoort, Sandro M. Krieg

**Affiliations:** 1 Department of Neurosurgery, TUM Neuroimaging Center, Technical University of Munich, Germany, School of Medicine, Klinikum rechts der Isar, Ismaninger Str. 22, 81675 Munich, Germany; 2 Centre for Language and Cognition Groningen (CLCG), University of Groningen, Groningen, the Netherlands; 3 Department of Neurosurgery, St Lucas Hospital, Ghent, Belgium; 4 Center for Language and Brain, National Research University Higher School of Economics, Moscow, Russia; 5 Department of Medical Rehabilitation, Federal Center for Cerebrovascular Pathology and Stroke, Moscow, Russia; 6 Department of Neurosurgery, Leeds Teaching Hospitals NHS Trust, Leeds, UK; 7 Department of Neurosurgery, Neurology, Brain Center Rudolph Magnus, University Medical Center Utrecht, Utrecht, the Netherlands; 8 Department of Neurosurgery, Lariboisière Hospital, APHP, Paris, France; 9 Department of Neurosurgery, Erasmus MC, University Medical Center, Rotterdam, the Netherlands; 10 Department of Neurosurgery, Hospital Garcia de Orta, Almada, Portugal

**Keywords:** Augmented reality, Awake surgery, Glioma, Tractography

## Abstract

**Background:**

The human white matter pathway network is complex and of critical importance for functionality. Thus, learning and understanding white matter tract anatomy is important for the training of neuroscientists and neurosurgeons. The study aims to test and evaluate a new method for fiber dissection using augmented reality (AR) in a group which is experienced in cadaver white matter dissection courses and in vivo tractography.

**Methods:**

Fifteen neurosurgeons, neurolinguists, and neuroscientists participated in this questionnaire-based study. We presented five cases of patients with left-sided perisylvian gliomas who underwent awake craniotomy. Diffusion tensor imaging fiber tracking (DTI FT) was performed and the language-related networks were visualized separated in different tracts by color. Participants were able to virtually dissect the prepared DTI FTs using a spatial computer and AR goggles. The application was evaluated through a questionnaire with answers from 0 (minimum) to 10 (maximum).

**Results:**

Participants rated the overall experience of AR fiber dissection with a median of 8 points (mean ± standard deviation 8.5 ± 1.4). Usefulness for fiber dissection courses and education in general was rated with 8 (8.3 ± 1.4) and 8 (8.1 ± 1.5) points, respectively. Educational value was expected to be high for several target audiences (student: median 9, 8.6 ± 1.4; resident: 9, 8.5 ± 1.8; surgeon: 9, 8.2 ± 2.4; scientist: 8.5, 8.0 ± 2.4). Even clinical application of AR fiber dissection was expected to be of value with a median of 7 points (7.0 ± 2.5).

**Conclusion:**

The present evaluation of this first application of AR for fiber dissection shows a throughout positive evaluation for educational purposes.

## Introduction

As we have learned from surgical and neuroscientific studies, knowledge of white matter anatomy is crucial for the surgical treatment of eloquent gliomas [3, 9, 14]. Preserving these structures is essential during the resection of eloquent brain tumors. Our current knowledge of this complex anatomy is currently based on cadaver dissections, insights through direct electrical stimulation (DES) during awake craniotomy surgery, and white matter tractography on MRI, for example by diffusion tensor imaging fiber tracking (DTI FT) [1, 4, 5, 7, 10, 13, 15]. Thus, learning and understanding white matter pathway anatomy is an important part of the training for neuroscientists and physicians. This is the motivation behind the growing number of white matter dissection courses being developed worldwide. However, these require costly infrastructure and brain anatomical specimens which can make such courses expensive and of only limited availability.

Advances have been made in the interface of digital image display and the usage of augmented reality (AR) devices in daily life. While long employed in gaming, prototype devices for neuroanatomical data visualization are now on the market. Through the use of specialized goggles combined with spatial computation, pre-processed imaging data can be visualized in an office, educational suite or conference room. Both in research and in clinical application, this new development has the potential to improve teaching and understanding of fiber tract anatomy. This system also has a potential advantage of making easier to identify white matter tracts which often remain difficult to identify during dissection of cadavers.

Ultimately, systems such as this could be of use in training neuroscientists and neurosurgeons, as well as for preparation and planning before surgery. While scrolling through the scans and viewing the anatomical objects in a group of clinicians, the surgical approach could be discussed in more detail with the full multidisciplinary team.

To date, the potential benefits of a virtual dissection device have not yet been evaluated reported. The objective of this study was to evaluate AR for the use in virtual fiber dissection, as well as assessing its potential application for virtual fiber dissection courses.

## Methods

### The European Low-Grade Glioma Network workshop meeting

The European Low-Grade Glioma Network (ELGGN) workshop group, consisting of 15 experts with neurosurgical, neurolinguistic, and neuroscientific experience, met in December 2019. Since the attending ELGGN members were experienced in cadaver and in vivo fiber dissections, we decided to evaluate AR and its potential for virtual fiber dissection courses with this target group.

### Prepared cases

The prepared cases consisted of patients who underwent microsurgical glioma resection during awake craniotomy at our department. Table 1 gives an overview on the patients and tumor characteristics as well as the clinical course. The study approved by the local ethics committee and was performed in accordance with the Declaration of Helsinki. All included patients provided written informed consent.

**Table 1:** Prepared cases

	Age	Tumor entity	Tumor hemisphere	Tumor location	Recurrent tumor	Prior resections	Awake resection	Language status		
								PreOP	POD5	POM3
Case 1	35	AA WHO °III	Left	Insular	Yes	Yes	Yes	1B	1B	1B
Case 2	38	OD WHO °II	Left	Parietal	No	No	Yes	0	0	0
Case 3	40	OD WHO °II	Left	Frontal	No	No	Yes	0	1B	0
Case 4	22	DA WHO °II	Left	Frontal	No	No	Yes	0	0	0
Case 5	58	GBM WHO °IV	Left	Parietal	No	No	Yes	0	1B	0

The table gives an overview on the prepared cases. *AA* anaplastic astrocytoma, *OD* oligodendroglioma, *DA* diffuse astrocytoma, *GBM* glioblastoma multiforme, *WHO* World Health Organization, *PreOP* preoperatively, *POD5* postoperative day 5, *POM3* postoperative month 3. Language grading: 0, no impairment of language function; 1, slight impairment of daily communication; 2, moderate impairment of language function, daily communication possible; 3, severe impairment of language function, daily communication not possible; A, non-fluent; B, fluent [12, 13]

### Technical setup

**Fig. 1:**
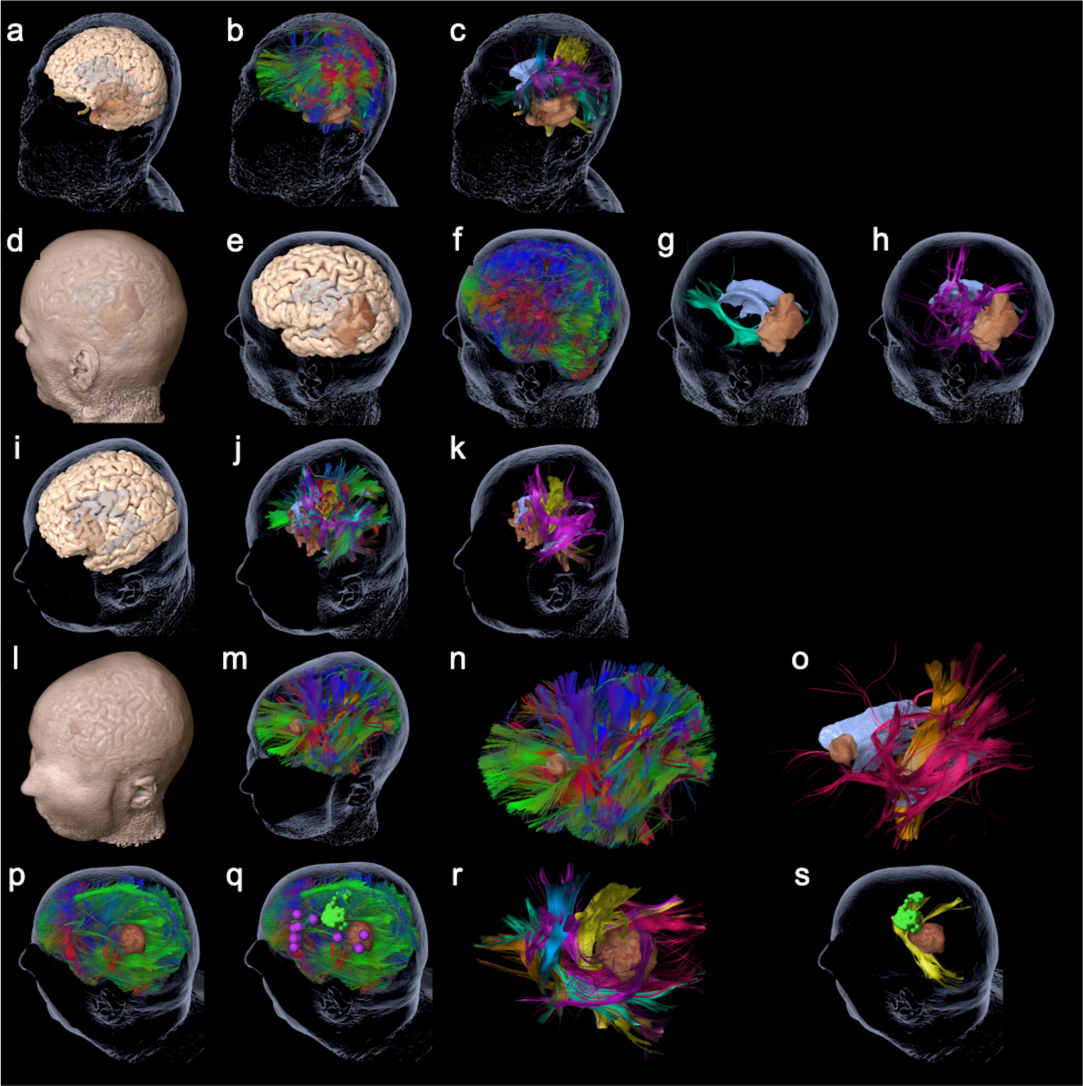
The figure shows the 3-D reconstructions of the prepared cases 1–5 (rows) as summarized in Table 1. Per row this figure shows the step by step reduction of anatomy until specific function-related fiber tracts are revealed. Case 1: **a** cerebral cortex and transparent skin including tumor and ventricles; **b** whole brain tractography; **c** specific fibers revealed, such as CST (yellow), FAT (blue), IFOF (green), and AF (pink). Case 2: **d** cerebral cortex and skin including tumor and ventricles; **e** cerebral cortex and transparent skin; **f** whole brain tractography; **g** IFOF (green), tumor, and ventricles; **h** SLF (pink), tumor, and ventricles. Case 3: **i** cerebral cortex and transparent skin including tumor and ventricles; **j** whole brain tractography; **k** specific fibers revealed for motor and language, such as CST (yellow) and SLF (pink). Case 4: **l** cerebral cortex and skin including tumor and ventricles; **m** whole brain tractography; **n** whole brain tractography without head; **o** specific language (pink) and motor (yellow)-related fibers revealed. Case 5: **p** whole brain tractography and skin including tumor and ventricles; **q** additional cortical location of motor (green) and language (pink) function; **r** specific fibers revealed, such as CST (yellow), FAT (blue), IFOF (green), SLF (pink), optic radiation (red), and tumor; **s** the skin, tumor, and ventricles plus MEP-positive sites of cortical motor function (green) with CST (yellow)

Firstly, the cortical surface and tractography of existing fiber bundles, based on commonly used clinical software, and delineation of other relevant regions of interest, such as a tumor to be resected, are required. The MRI sequences which were used for the DTI FT were performed on 3 T magnetic resonance scanners (Philips Medical System, Netherlands B.V.). All patients obtained MRIs according to the standard glioma protocol at our department including a T1-weighted three-dimensional (3-D) gradient echo sequence with intravenous contrast administration for anatomical co-registration, a T2-weighted 3-D FLAIR sequence, and DTI sequences with 32 orthogonal sequences. For the visualization of white matter pathways, we performed DTI FT of eloquent pathways such as the corticospinal tract (CST), the arcuate fasciculus (AF), the inferior and middle fronto-occipital fasciculus (IFOF, MFOF), the frontal aslant tract (FAT), and the superior and inferior longitudinal fasciculus (SLF, ILF). Therefore, we used our standard deterministic algorithm with a fiber assignment by continuous tracking (FACT) (iPlan® Net Cranial 3.0.1 and Brainlab Elements, Brainlab AG, Munich, Germany). The regions of interest (ROI) were chosen based upon anatomy and function. The latter ROIs were based on preoperative navigated transcranial magnetic stimulation mappings (nTMS) [12]. Figure 1 shows the 3-D reconstructions and the visualized steps of the prepared cases.

Here, the special feature for fiber dissection was the opportunity for participants to virtually dissect prepared white matter pathways and objects by the use of a spatial computer and AR goggles (Brainlab AG, Munich, Germany; Magic Leap Inc., Plantation, Florida, USA). In contrast to a virtual reality setup, AR allows to still see the room and other participants while the 3-D objects are projected inside the actual environment.

### Augmented reality fiber dissection

**Fig. 2:**
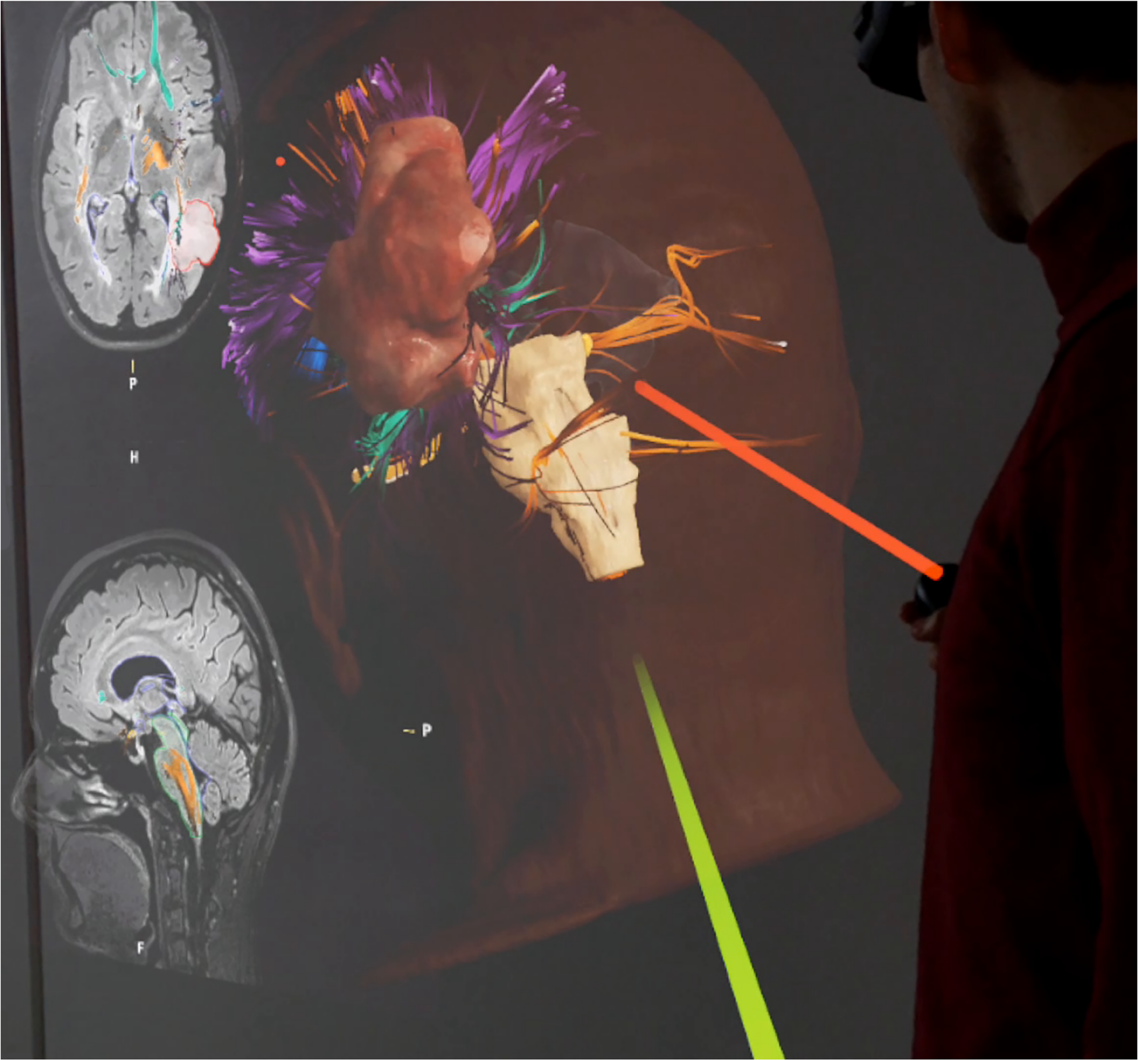
The figure shows a screenshot visualizing the participant view during the application of AR for fiber dissection. The 3-D reconstruction is shown from left occipital. The green and red laser pointers can be used to describe structures or answer questions between participants or moderator. Additionally, the patient’s 2-D MRI scan slices can be visualized in the background and can be scrolled

**Fig. 3:**
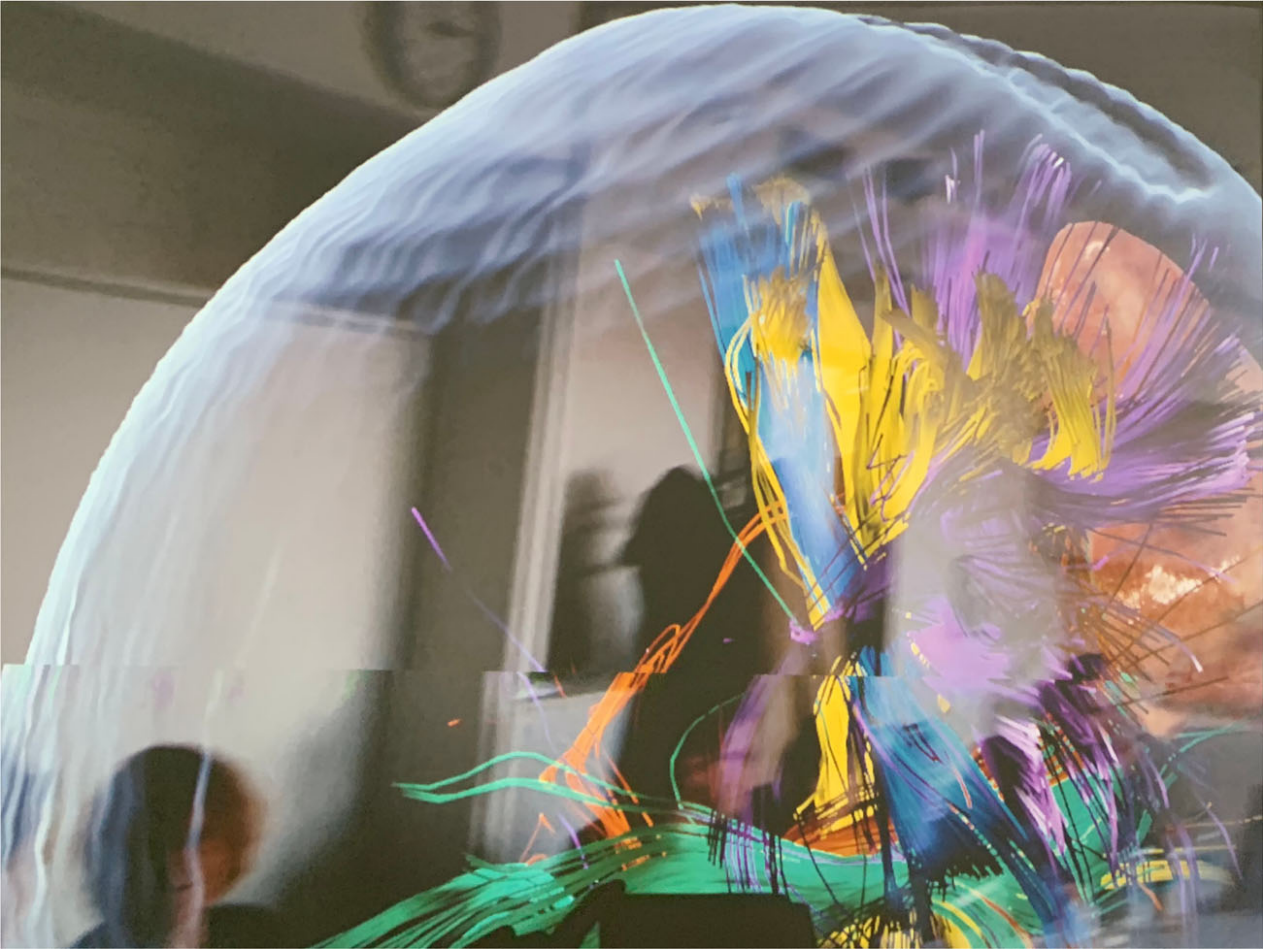
The figure shows another screenshot visualizing the participants view during the application of AR for fiber dissection. The different colors show different tracts. The tractography model is projected in the room

A 3-D model of the chosen objects can be added into the AR display (Figs. 2 and 3). The pre-selected cases were presented chronologically (Fig. 1). The participants were divided into subgroups of five. Each participant was equipped with an AR goggle headset. In each subgroup of five, one was designated as “master.” The master was equipped with a controller which allowed them to zoom and rotate the model. They were also able to perform virtual dissection of the fiber tracts by peeling away additional layers. In additional, the master’s view was presented via live screen for review by the remainder of the group who were not wearing goggles for that particular session. Figures 2 and 3 show screenshots of the participant view during the application of AR for fiber dissection. Figure 1 shows the step-by-step neuroanatomy dissection revealing function-related fiber tracts.

### Questionnaire

To evaluate the application of AR for fiber dissection, participants were asked to complete a questionnaire on their normal use of tractography, as well as this simulation experience of using AR goggles and a spatial computer for fiber dissection. They were also asked about possible future applications and suggestions for improvement (Table 2).

**Table 2:** Answers on questions 1–14

Questions												Participants	Answers	Median	Range		Mean		SD	
Question 1: Do you use tractography at your institution—0 = never, 10 = routinely?																				
Answers	0	1	2	3	4	5	6	7	8	9	10									
Number	0	0	0	2	0	4	1	0	2	1	5	15	15	8	3–10		7.1		2.6	
%	0	0	0	13.3	0	26.7	6.7	0	13.3	6.7	33.3									
Question 2: What is the major application of tractography at your institution?																				
Answers	None		Only research		Only clinical		Both													
Number	0		5		3		7					15	15	-	-		-		-	
%	00		33.3		20.0		46.7													
Question 3: Do you trust in tractography—0 = not at all, 10 = completely?																				
Answers	0	1	2	3	4	5	6	7	8	9	10									
Number	0	0	0	0	1	3	2	4	5	0	0	15	15	7	4–8		6.6		1.3	
%	0	0	0	0	6.7	20.0	13.3	26.7	33.3	0	0									
Question 4: Which regions of interest do you use for tractography (multiple answers possible)?																				
Answers	Anatomy-based		Atlas-based		Function-based															
Number	10		4		6							15	20	-	-		-		-	
%	66.7		26.7		40.0															
Question 5: How do you rate your overall experience of augmented reality for fiber dissection—0 = boring, 10 = exciting?																				
Answers	0	1	2	3	4		5	6		7		8	9	10						
Number	0	0	0	0	0		1	0		2		5	2	5	15	15	8	5–10	8.5	1.4
%	0	0	0	0	0		6.7	0		13.3		33.3	13.3	33.3						
Question 6: Do you think augmented reality for fiber dissection could be a useful addition to fiber dissection courses—0 = not at all, 10 = absolutely?																				
Answers	0	1	2	3	4		5	6		7		8	9	10						
Number	0	0	0	0	0		1	1		1		6	2	4	15	15	8	5–10	8.3	1.4
%	0	0	0	0	0		6.7	6.7		6.7		40.0	13.3	26.7						
Question 7: How do you rate the usefulness of augmented reality for fiber dissection for education in general—0 = not at all, 10 = very useful?																				
Answers	0	1	2	3	4		5	6		7		8	9	10						
Number	0	0	0	0	0		1	2		0		7	1	4	15	15	8	5–10	8.1	1.5
%	0	0	0	0	0		6.7	13.3		0		46.7	6.7	26.7						
Question 8: For which qualification would you use education by augmented reality for fiber dissection—0 = not useful, 10 = very useful?																				
Student																				
Answers	0	1	2	3	4		5	6		7		8	9	10						
Number	0	0	0	0	0		1	0		2		3	4	5	15	15	9	5–10	8.6	1.4
%	0	0	0	0	0		6.7	0		13.3		20.0	26.7	33.3						
Resident																				
Answers	0	1	2	3	4		5	6		7		8	9	10						
Number	0	0	0	0	1		1	0		0		4	3	6	15	15	9	4–10	8.5	1.8
%	0	0	0	0	6.7		6.7	0		0		26.7	20.0	40.0						
Surgeon																				
Answers	0	1	2	3	4		5	6		7		8	9	10						
Number	0	0	1	0	1		0	0		1		3	2	6	14	14	9	4–10	8.2	2.4
%	0.000	0.000	0.071	0.000	0.071		0.000	0.000		0.071		0.214	0.143	0.429						
Scientist																				
Answers	0	1	2	3	4		5	6		7		8	9	10						
Number	0	0	1	0	1		0	1		0		4	2	5	14	14	8.5	2–10	8.0	2.4
%	0	0	7.1	0	7.1		0	7.1		0		28.6	14.3	35.7						
Question 9: Do you think there is a value for clinical application of augmented reality for fiber dissection—0 = not at all, 10 = absolutely?																				
Answers	0	1	2	3	4		5	6		7		8	9	10						
Number	1	0	0	0	0		3	0		5		2	1	3	15	15	7	0–10	7.0	2.5
%	6.7	0	0	0	0		20.0	0		33.3		13.3	6.7	20.0						
Question 10: For which clinical application could augmented reality for fiber dissection be used in future (multiple answers possible)?																				
Answers	Resident		PreOP		IntraOP			Patient consultation												
Number	8		14		3			4							15	29	-	-	-	-
%	53.3		93.3		20.0			26.7												
Question 11: Do the presented cases reflect your clinical reality?																				
Answers	Yes		No																	
Number	12		1												13	13	-	-	-	-
%	92.3		7.7																	
Question 12: Are the visualized fiber tracts anatomically correct?																				
Answers	Yes		No																	
Number	6		4												10	10	-	-	-	-
%	60.0		40.0																	
Question 13: Did you see a difference between anatomy-based and function-based tractography?																				
Answers	No		Yes favoring anatomy		Yes favoring function															
Number	2		2		8										12	12	-	-	-	-
%	16.7		16.7		66.7															
Question 14: Is DTI enough augmented reality for fiber dissection or would you recommend using a more sophisticated approach for such fiber dissection?																				
Answers	Enough		More sophisticated																	
Number	9		5												14	14	-	-	-	-
%	64.3		35.7																	

The table shows the results of questions on the participants’ use of tractography at their institutions and on the participants’ impression of the AR fiber dissection session

## Results

### Participant’s experience

Table 2 summarize the overall experience, the usefulness for fiber dissection courses, and the value for educational and clinical purposes of AR for fiber dissection as rated by the participants (Table 2).

### Suggestions for future improvements

After the general evaluation of AR fiber dissection by questions 1–14, we also asked for suggestions for future improvements. The participants’ answers on the open questions 15 (“What should be improved before augmented reality for fiber dissection can be used for virtual fiber dissection?”) and 16 (“What should be improved before augmented reality for fiber dissection can be used for clinical purposes?”) are incorporated in the “Discussion” section of this manuscript.

## Discussion

### Augmented reality for fiber dissection

The described setup in this study currently is the only approach allowing for an augmented reality illustration of real-life cases in an easy and straightforward way not requiring repeated rendering and multistep workflows [11]. While AR was already described extensively in other surgical applications, spatial computing allowing for an accurate and real-time visualization of white matter anatomy was only feasible recently [8], yet as an overlay via microscope integration, neurosurgeons know AR for some years [2, 6].

Overall, the participants in this study of AR fiber tract dissection felt that this was a positive development with the potential for valuable educational and clinical benefits in the future.

As shown by the results of question 1–4, meanwhile, tractography is standardly used for clinical and scientific purposes (Table 2). Moreover, proven by the composition of the participants, various disciplines frequently use this technique for the visualization of white matter pathways, and 92.3% of participants evaluated the presented cases as reflecting their clinical reality. Hence, the present results of the evaluation of AR for fiber dissection as a new technique for visualization are of high relevance for learning, teaching, discussing, and understanding of both the methods for tractography and anatomy.

As was evident from both the individual reactions during the evaluation, as well as the formal questionnaire, the participants were excited and impressed by the technology (questions 5–8, Table 2). Based on the survey responses, the primary applications were felt to be for the educational and training of students, residents, and junior scientists. In addition, the relevance for board-certified neurosurgeons was highly rated, with a median score of 9 of 10 (Table 2).

As expected at the outset of the study, the clinical applicability of AR fiber dissection is restricted at the moment. This was confirmed by the participants. However, 14 of 15 participants said they felt that this AR technique could be developed for clinical settings as a tool for pre-operative preparation of the respective surgeon (Table 2).

### Improvement suggestions

Questions 15 and 16 were open questions, allowing participants the opportunity to make suggestions for improvements to the presented technique. Their answers were mainly related to the interaction of the user with the spatial computer, the goggles, and the options of visualization.

Participants recommended that, for future applications, more anatomical structures should be included in the simulation to improve orientation. In fact, this is already available but was not offered as an option for this simulation assessment. The anatomical reconstructions used included pre-selected language pathways, the 3-D reconstruction of the tumor, and the modeling of the patients’ skin (Fig. 1). We also included a reconstruction of the cortex to evaluate this additional request (Fig. 1).

For this study, we used data from patients with brain tumors to demonstrate the virtual dissections in these patients. The participants also suggested that including fiber tracts from healthy subjects would be helpful to facilitate greater understanding of normal anatomy. We feel that a particular strength of the virtual approach is that we are able to use AR to dissect both disease-free normal anatomy and actual tumor cases, improving our experience and understanding of tumor-related changes to normal anatomy in a manner which is not possible in cadaver courses.

The participants also suggested that it would be helpful to develop a more individualized interactivity, which could allow each individual user to control the peeling (virtual dissection), zooming, and rotating of the reconstruction. Likewise, participants asked for development of the ability to separately include and exclude single pathways and to label the fiber tracts with their name and function, either as a default setting or after their identification. During the simulation and assessment session, participants were individually able to walk through the reconstruction. However, functions like zooming, rotating, and the inclusion/exclusion of structures could only be performed by the single master controller, who was the only enabled interactor. We chose this format to facilitate a clearer presentation. However, the currently version of the software already enables all participants to operate individual controllers, so the limitation experienced by the participants was simply due to the setup for the assessment trial.

Another suggestion for future development was to enable the user to interact with the reconstruction with their hands, without needing a controller. Some participants also asked for a wider field-of-view in the goggles, to improve orientation, especially during zooming and rotating of the reconstruction.

In summary, the participants felt that the overall AR system was useful and had good potential for development and a wide range of potential future applications in education and clinical settings. The requested changes are useful for improving the usability of the system in the future.

### Limitations and evaluation of tractography

This report only aimed to evaluate a new AR technique for fiber dissection but not the different methods and algorithms for tractography. However, the latter is frequently discussed, so we sought to evaluate this through questions 12–14. As reflected by the answers and through discussions during the meeting, the actual tractography techniques are also an issue when evaluating techniques for fiber tract visualization. The visualized fiber tracts were rated as anatomically correct by 60% and as incorrect by 40%, which must also be seen as a major limitation of tractography. Most of the participants (66.7%) favored the setting of function-based ROIs and 16.7% of participants did not see a difference between anatomy-based and function-based ROIs. Moreover, while 64.3% of participants felt that DTI FT was sufficient for the presented approach of fiber dissection, 35.7% of participants asked for a more sophisticated approach for future applications. The variation of opinion regarding tractography algorithms and methods could be seen as a limitation of this initial study. However, these issues can be addressed with relative ease and speed, so we do not feel that it is a significant limitation for future development of the technique itself.

Apart from these limitations, here, it must also be mentioned that further options might exist for the visualization of fiber tracts in future, for example smartphone-based solutions.

## Conclusion

This study evaluating the application of AR for fiber dissection found a generally positive response as rated by different complementary neuroscience specialties. Most participants felt that the greatest initial application would be for educational purposes, with potential clinical uses dependent upon the future developments of the system.
